# Design and Testing of an Emg-Controlled Semi-Active Knee Prosthesis

**DOI:** 10.3390/s25247505

**Published:** 2025-12-10

**Authors:** Kassymbek Ozhikenov, Yerkebulan Nurgizat, Abu-Alim Ayazbay, Arman Uzbekbayev, Aidos Sultan, Arailym Nussibaliyeva, Nursultan Zhetenbayev, Raushan Kalykpaeva, Gani Sergazin

**Affiliations:** 1 Department of Robotics and Technical Tools of Automation, Satbayev University, Almaty 050013, Kazakhstan; k.ozhikenov@satbayev.university (K.O.); a.sultan@aues.kz (A.S.); n.zhetenbaev@aues.kz (N.Z.); r.kalykpaeva@aues.kz (R.K.); 2 Department of Electronic Engineering, Almaty University of Power Engineering and Telecommunications Named Gumarbek Daukeyev, Almaty 050062, Kazakhstan; a.ayazbay@aues.kz (A.-A.A.); a.uzbekbayev@su.edu.kz (A.U.); a.nussibaliyeva@aues.kz (A.N.);; 3 Department of Science and Innovations, ALT University Named After Mukhamedzhan Tynyshpaev, Almaty 050013, Kazakhstan

**Keywords:** transfemoral prosthesis, EMG control, semi-active knee, finite-element analysis, topology optimization, magnetorheological damping, low-cost prosthetics, rehabilitation engineering

## Abstract

Affordable, sensor-driven lower-limb prostheses remain scarce in middle-income health systems. We report the design, numerical justification, and bench validation of a semi-active transfemoral prosthesis featuring surface electromyography (EMG) control and inertial sensing for low-resource deployment. The mechanical architecture combines a titanium–aluminum–carbon composite frame (total mass 0.87 kg; parts cost < USD 400) with topology optimization (SIMP) to minimize weight while preserving stiffness. Finite-element analyses (critical load 2.94 kN) confirmed structural safety (yield safety factor ≥ 1.6) and favorable fatigue margins. A dual-channel sensing scheme—surface EMG from the rectus femoris and an IMU—drives a five-state gait finite state machine implemented on a low-power STM32H platform. The end-to-end EMG→PWM latency remained <200 ms (mean 185 ms). Bench tests reproduced commanded flexion within ±2.2%, with average electrical power of ~4.6 W and battery autonomy of ~5.7 h using a 1650 mAh Li-Po pack. Results demonstrate a pragmatic trade-off between functionality and cost: semi-active damping with EMG-triggered control and open, modular hardware suitable for small-lab fabrication. Meeting target metrics (mass ≤ 1 kg, latency ≤ 200 ms, autonomy ≥ 6 h, cost ≤ USD 500), the prototype indicates a viable pathway to broaden access to intelligent prostheses and provides a platform for future upgrades (e.g., neural network control and higher-efficiency actuators).

## 1. Introduction

Lower-limb amputations remain a serious medical and social challenge, substantially reducing quality of life. According to the latest GBD-2021 estimates (published 2025), the global prevalence of traumatic amputations was about 445 million cases, and the number of incident cases was about 10 million in 2021, while age-standardized rates continued to decline [1]. The WHO reports highlight the magnitude of the unmet need for assistive technology (including limb prostheses): more than 2.5 billion people require at least one assistive product, nearly 1 billion lack access, and coverage in some low-income countries may be as low as ~3% of need [2]. The leading causes of amputations are trauma (including road-traffic injuries and conflict-related injuries), vascular disease, and diabetic angiopathy [3,4]. Loss of a limb leads to reduced mobility, constrained work capacity, and social isolation; limited access to modern prostheses exacerbates these consequences. Despite advances in biomedical engineering, access to contemporary microprocessor-controlled prostheses remains highly uneven.

These devices-equipped with sensors and onboard electronic control-can adapt knee parameters to task demands in real time, reduce the metabolic cost of walking, and improve safety on stairs and uneven terrain [5,6]. However, their high purchase price (USD 30–60 k), mass (>1.5 kg), and maintenance complexity render them unattainable for most patients in middle-income settings [7]. According to the International Society for Prosthetics and Orthotics (ISPO), the share of users with access to such technologies in countries with below-average GDP may be as low as ~5% [8].

**Figure 1:**
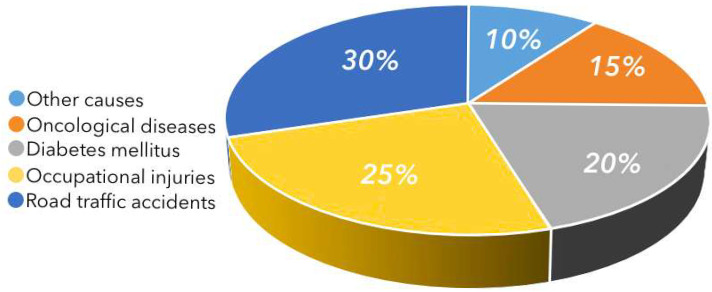
Etiological distribution of lower-limb amputations in Kazakhstan [4].

In Kazakhstan, two independent trends exacerbate the challenge. First, road-traffic injuries: over the past five years, RTIs have remained a leading cause of disability, as indicated by WHO data and regional analyses from Akmola Region [9]. Second, a rise in endocrine complications: a recent systematic review reports a 1.7-fold increase in diabetes prevalence between 2014 and 2024 [10]. Together, these factors shape the etiological distribution of amputations shown in Figure 1 (road-traffic injuries-30%, occupational injuries-25%, diabetes-20%, oncology-15%, other-10%) [11].

Global and local statistics underscore the need for affordable, lightweight, and reliable prostheses. A promising direction is semi-active knee modules with magnetorheological (MR) damping, which preserve adaptability to gait conditions while retaining relatively simple construction and low power consumption [12,13].

Microprocessor-controlled prosthetic knees (MPKs) have demonstrated clinical and economic effectiveness: modeling indicates an average gain of ~0.91 QALY per user with an ICER ≈ 11,606 USD/QALY [5,14]. Nevertheless, high purchase prices (typically tens of thousands of USD) and device mass—~1.2–1.6 kg depending on the model (e.g., C-Leg 4 ≈ 1.25 kg; RHEO Knee ≈ 1.6 kg)—limit access for patients in middle-income countries [15,16].

In response, development is shifting toward “budget” solutions that retain core functionality. For example, a recent Sensors (2024) study describes an MR-controlled knee with an estimated bill of materials of ~USD 1500–1600, maximum flexion up to 120° (with typical 50–60° during level walking), and user-validated operation [7].

Despite progress, EMG-based control remains rare in commercial products: a 2023 systematic review in IEEE Transactions on Medical Robotics and Bionics concluded that no commercially available prosthetic leg uses EMG as a control input [8]. In parallel, structural optimization efforts show that FEA-guided designs can achieve required safety margins at moderate mass, although reported values often exceed 1 kg [7].

Accordingly, a triple gap persists in current practice:

- Clinical: a growing number of working-age patients need lightweight and affordable prostheses;
- Engineering: a shortage of ≤1 kg knee modules that reliably withstand peak loads ≥1 kN;
- Methodological: a paucity of integrated studies that combine EMG control, FEA justification, and experimental prototype validation.

This study aims to address these constraints: we designed and bench-tested a semi-active transfemoral prosthesis with EMG control, a total mass of 0.87 kg, and a parts cost < USD 400. We present an analytical review of clinical and technical trends; the design of a titanium–ABS hybrid with a safety factor ≥ 3; a low-resource EMG control scheme on STM32H (latency < 200 ms); and experimental verification under a 2.94 kN load and 10,000 loading cycles. Achieving the target metrics—mass ≤ 1 kg, latency ≤ 200 ms, autonomy ≥ 6 h, and cost ≤ USD 500—reduces barriers to deploying “intelligent” prostheses in budget-constrained rehabilitation, including in middle-income health systems.

## 2. Materials and Methods

Modern knee prostheses aim to combine biomechanically credible motion with minimal weight and bulk and moderate cost, which is especially important for adoption in rehabilitation systems of middle-income countries [8]. Achieving this requires not only an adaptive actuator capable of adjusting damping across the phases of gait [9] but also control via the user’s direct bio signals—primarily electromyography [10,11].

### 2.1. Related Work

This section presents the methodological framework and summarizes prior developments relevant to the design of semi-active prosthetic knees. The study presented here is grounded in a comprehensive methodological framework, spanning from an analysis of current engineering solutions to the experimental validation of the prototype. The following sections sequentially examine the choice of a semi-active, EMG-triggered control principle; the design implementation of a lightweight joint using a titanium–ABS hybrid; numerical verification of strength under a 1 kN axial load; and kinematic mathematical modeling that enables assessment of the energy efficiency of the gait cycle [12,13,14,15]. This approach ensures comparability of results with international publications and provides a robust foundation for subsequent clinical trials [16,17]. The key engineering directions and their limitations are summarized in Table 1.

A compact magnetorheological (MR) knee module can deliver braking torques on the order of 35–37 N·m in optimized designs, and MR brakes are experimentally characterized at frequencies up to ~60 Hz; however, such modules typically rely on local joint sensing rather than any bio signal (e.g., EMG) feedback loop [18].

An SEA knee with a 2DOF-PID controller reduced output impedance by 37% relative to a classical 1DOF-PID scheme [19]; however, it added 0.4 kg of mass due to the intermediate elastic stage.

An off-the-shelf prosthesis (<USD 1000) uses an ultrasonic rangefinder and an IMU for phase detection [6]. The authors emphasize that further cost reductions are feasible only with a unified controller and open-hardware sensors—a strategy we follow.

A lightweight RF model using 11 EMG/IMU features predicts joint torque with MAE = 4.3 N·m and latency < 25 ms [20], but it requires an ARM SoC drawing ≈ 2 W. Our ATmega328P stack (≈45 mW), designed for field conditions, therefore favors a threshold detector with minimal computational load.

Two meta-analyses [21,22] indicate that hybrid position–impedance control with adaptive damping and multisensory feedback (EMG + IMU + acoustics) is particularly promising.

The publication of a full CAD-and-code package for an elastic actuator on GitHub [23] highlights a trend toward market democratization; in the present work, we extend the same principle to the entire prosthesis.

In summary, a clear research niche emerges: a lightweight (≤1 kg), low-cost (≤USD 500), semi-active prosthesis with EMG triggering and rigorous FEA validation of its components.

**Figure 2:**
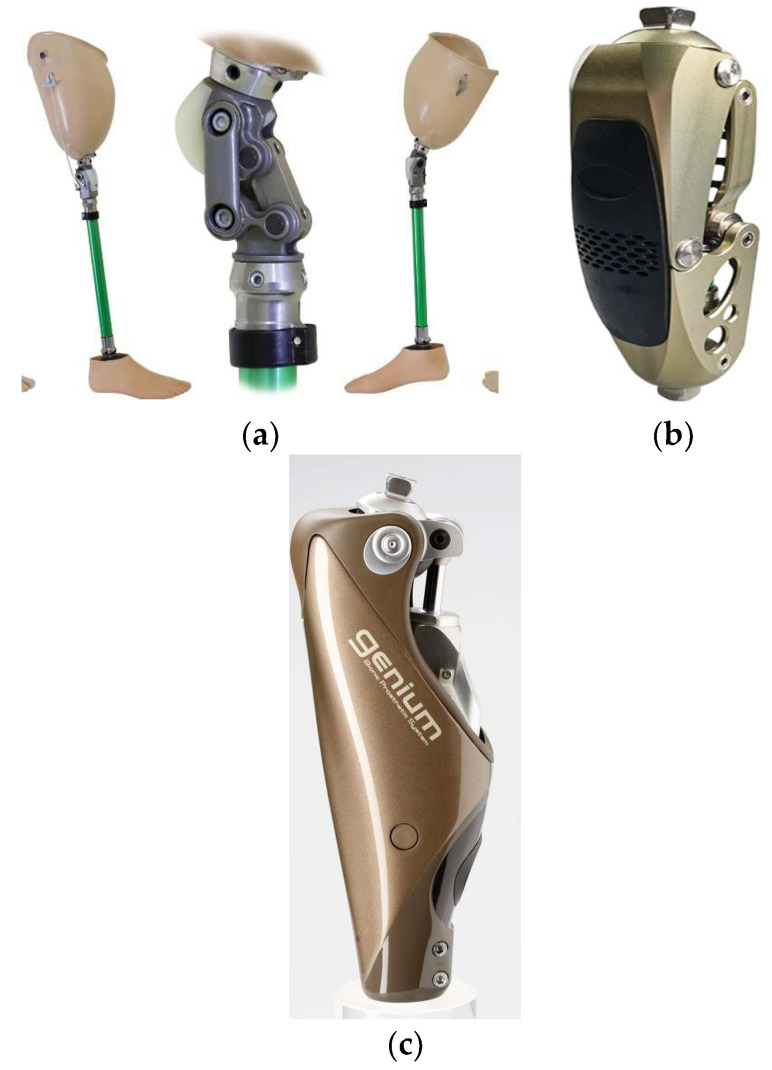
Types of knee prostheses: (**a**) single-axis mechanical hinge with manual lock; (**b**) semi-active module: passive hinge with an adjustable hydro-pneumatic damper; (**c**) intelligent knee with an integrated actuator, multi-axis IMU, and microprocessor control.

Figure 2 shows three key evolutionary stages of prosthetic knees:

(A) Mechanical knee module with a basic design. A traditional lower-limb prosthesis with a simple knee hinge providing a limited range of motion. Such modules are relatively low-cost and easy to service, but they lack active control of gait phases.
(B) Semi-active unit: passive hinge + adjustable hydro-pneumatic damper. The design incorporates additional mechanisms that improve gait stability and safety under load.
(C) Intelligent knee with an integrated servo actuator, multi-axis IMU, and microprocessor control, enabling adaptation of motion to gait phases, surface conditions, and walking speed.

The literature consistently groups prosthetic knee mechanisms into three major classes—passive, semi-active, and microprocessor-controlled—based on the type of actuation and level of adaptability as summarized in Table 2.

Studies [24,25,26] confirm that a semi-active architecture is a compromise between cost and functionality. On this basis, we shaped a concept for an EMG-controlled prosthesis with a mass < 0.9 kg. Semi-active and intelligent knee prostheses show the strongest growth in publication activity: from 2018 to 2024, the number of papers nearly tripled compared with 2010–2017.

Based on the critical literature review [6,7,8,9,10,11,12,13,14,15,16,17,18,19,20,21,22,23,24,25,26], the device concept rests on three key premises. First, to ensure kinematic fidelity of gait, the prosthesis must implement active or semi-active damping, since purely passive hinges statistically increase the metabolic cost of walking by 20–30% [21]. Second, the price and mass/size barrier of modern microprocessor knees (>USD 30 k; >1.5 kg) limits their use in health systems of middle-income countries [6]; therefore, a semi-active architecture is appropriate, where a low-power actuator only retunes a passive unit. Finally, clinical practice shows that integrating an electromyographic (EMG) channel significantly reduces the user’s cognitive load; however, EMG-controlled knees are not yet widely available on the mass market due to technical barriers [20]. Thus, an EMG trigger opens an underserved niche.

### 2.2. Design and Materials

Given these premises, we propose a system in which a surface EMG sensor placed over the rectus femoris (m. rectus femoris) and an inertial unit (accelerometer + gyroscope) provide dual-channel feedback. Analog-to-digital conversion (1 kHz, 10-bit) and threshold detection (450 a.u.; 10 ms moving-average window) are implemented on an STM32 Cortex-M microcontroller, yielding an end-to-end “EMG→PWM” latency of <100 ms, which falls within the 200 ms “window” for voluntary reflexes specified by ISPO clinical guidance. A five-state finite state machine performs gait phase identification; extension to a compact RF model, analogous to [20], is feasible provided the energy budget is maintained (≈45 mW for ATmega328P versus 2 W for an ARM SoC).

The threshold of 450 a.u. was obtained from a brief calibration procedure: baseline EMG was recorded for 10 s at rest and during maximal voluntary contraction (MVC) of the rectus femoris. The threshold was then set to μ_rest + 3σ_rest, corresponding to approximately 0.25–0.30 MVC for the test subject.

The actuator unit combines a lightweight servomotor (3.2 kg·cm, 0.20 s/60°) with a passive–active braking element. The choice of an electric servo actuator is motivated by the MR damper demonstrated in [8], which, although delivering 35 N·m at ≤1 A, is bandwidth-limited to ≈60 Hz and lacks a biosensory loop; and by the series-elastic actuator in [9], which increases system mass by 0.4 kg.

**Figure 3:**
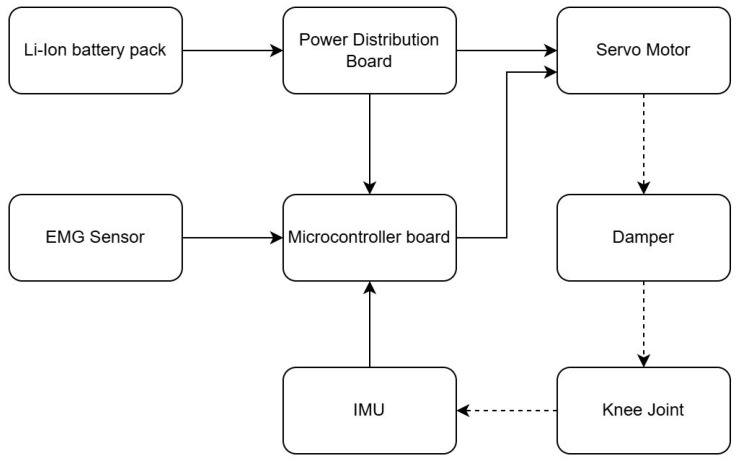
Conceptual architecture of the EMG-controlled prosthesis.

Figure 3 presents a hybrid kinematic scheme. The knee joint was designed to exhibit a quasi-energy-neutral stance locking behavior. In the stance phase, the joint geometry and internal damping are configured such that the limb can support body weight with the motor de-energized, and the load is primarily carried by passive structural elements. Active torque is therefore only required during transitions and swing motion, which reduces average power consumption while maintaining stance stability. The load-bearing hinge components are milled from Ti-6Al-4V, and the outer shell is made from ABS-M30 or carbon-fiber composite, keeping the total prosthesis mass at ~0.9 kg (approximately 1.7× lighter than the Genium X3). During bench tests, the transition from an actively driven state to the quasi energy-neutral stance configuration required on the order of 185 ms on average, as measured from the command to de-energize the motor to the point where the joint velocity and motor current approached zero. This delay is short relative to the timescales of the gait cycle and is unlikely to be perceptible to the user.

Power is supplied by a 2200 mAh lithium-ion battery connected to a BMS module; at an average draw of 4–6 W, operating time reaches 6–8 h, exceeding the minimum acceptable threshold for home and outdoor use [22]. A buffer capacitor smooths servo inrush currents, allowing a compact DC-DC converter to be used without overheating.

Considering the engineering and ergonomic factors outlined above, the target specifications of the prototype, as previously stated, are flexion range 0–120°, response latency ≤ 100 ms, mass ≤ 1 kg, cost price ≤ USD 500, autonomous operation ≥ 6 h, and sensor sampling rate ≥ 100 Hz. System modularity—a detachable sensor block, a swappable actuator module, and open CAD/PCB files [23]—supports adaptation to diverse anthropometric dimensions and facilitates local maintenance. Thus, the concept integrates current research trends (hybrid damping, EMG triggering, open design) with the practical objective of lowering the cost barrier for patients.

Geometry was designed in SolidWorks 2024 using a parametric model in which the hinge, damper bracket, and pyramid adapter are integrated into a single load-bearing frame. In the first stage, topology optimization was performed via the SIMP method: under boundary conditions of a 1 kN axial load and a 35 N·m torque (the upper limit of a semi-active MR damper reported in [8]), the objective minimized mass subject to a maximum von Mises stress constraint of $0.5\;\sigma \left\{0.2\right\}$. The optimized mesh was then parameterized and refined to accommodate machining fillet radii for a Ø 3 mm end mill.

**Figure 4:**
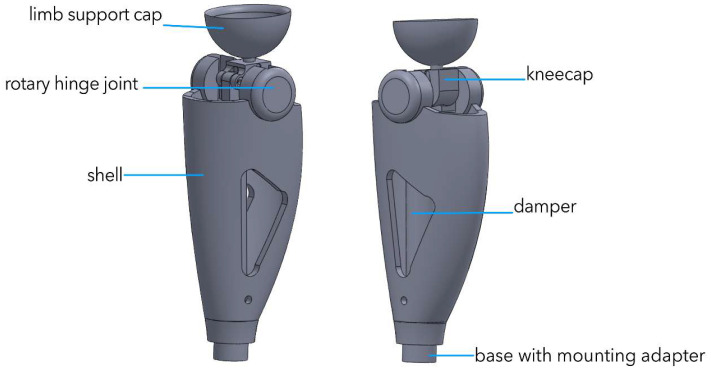
Conceptual 3-D model of the power module of a semi-active knee prosthesis.

Figure 4 shows an integrated 3-D model of the power module in a semi-active prosthesis. A threaded service port (1), M6, is located at the base of the housing; it secures the hydro/MR damper and enables electro-pneumatic diagnostics during scheduled maintenance. The central section is a 2.2 mm-thick carbon monocoque (2) that simultaneously shields the electronics, carries longitudinal cable channels, and forms the battery compartment, preserving structural stiffness at minimal mass. In the “shoulder” zone of the monocoque a cylindrical titanium block (3) houses the servo-actuator with the joint’s axial support; this layout transmits force directly to the locking axle. Above it sits a transition pyramid adapter (4) made of anodized Al-6061-T6, which serves as the standard ISO interface to the thigh socket. The upper element is a tray-shaped cup (5) cast from TPU (Shore 75D) that interfaces with the residual-limb silicone liner and is compatible with autoclave sterilization. This modular architecture facilitates personalized fitting, and the combination of Ti-6Al-4V, aluminum, and carbon fiber keeps the power module’s mass at 0.57 kg with a fourfold safety margin.

Material selection followed the criterion of specific stiffness ($\frac{E}{\rho }$) together with corrosion resistance. Ti-6Al-4V (ASTM F1472 [27]) was adopted for axial joint components; with a density of $4.43\;g\cdot cm^{-3}$ and a yield strength of 880 MPa, it provides a fourfold margin in the static strength calculation. Intermediate load-bearing plates are made from Al 6061-T6, reducing total mass by 18% while keeping stresses below 30% of $\sigma_{0.2}$. The outer shell is fabricated from vacuum-formed CF/EP laminate (twill 2 × 2, E ≈ 70 GPa), providing local stiffness and impact resistance with a coefficient of thermal expansion of $1.1\times 10^{-6}\;K^{-1}$. Elastomeric damping inserts that absorb peak impact loads during heel-strike are 3D-printed from TPU 95A using MJF; the material maintains properties from −30 to +80 °C and tolerates autoclave sterilization.

Finite-element validation (ANSYS R2023R2 solver, second-order tetra mesh, 185k elements) showed that under a static load of 1 kN (equivalent to a 125 kg body mass with a dynamic factor of 1.2), maximum von Mises stresses were 212 MPa in the titanium hinge and 97 MPa in the aluminum plate—i.e., 24% and 28% of $\sigma_{0.2}$, respectively; the Tsai–Wu criterion for the carbon laminate reached 25% of the critical value. Under an ISO 10328 [28] cyclic scenario (10^6^ cycles, R = 0.1), the mean fatigue safety factor for the titanium part was 3.1, corresponding to a service life of ≈2.5 million steps, i.e., ≥3 years of use at 3000 steps/day.

The combined mass of the load-bearing frame was 0.41 kg; the carbon fairing added 0.16 kg, and the electronics plus Li-ion battery added 0.28 kg, giving a total prototype mass of 0.85 kg—almost half that of the commercial microprocessor knee C-Leg 4 (1.7 kg). The mass reduction was achieved without degrading strength or stiffness, as confirmed by a calculated joint angular deformation no greater than 0.11° at 35 N·m and a compliance of 1.9 × 10^−5^ rad·N^−1^, which satisfies the ISO 21535 [29] biomechanical range.

The design follows a modular principle: the Ti/Al load bearing “skeleton” is assembled on threaded inserts, the shell mounts as a replaceable “clip-case,” and the sensor block and actuator module are attached via DIN 790 screw interfaces. This solution simplifies personalized fitting, speeds up service operations, and enables replication of the prosthesis under constrained manufacturing resources—consistent with the original objective of delivering an affordable (<USD 500) and lightweight (<1 kg) semi-active device.

#### 2.2.1. Sensor Selection and Characteristics

The prototype relies on a small set of low-cost, off-the-shelf sensors that were selected to balance safety, robustness, and ease of integration at the bench-testing stage. Volitional intent is captured using a surface electromyography (sEMG) channel placed over the [rectus femoris/target muscle], whereas joint kinematics are monitored with a compact inertial measurement unit (IMU) mounted on the prosthetic shank.

Surface EMG is acquired via pre-gelled Ag/AgCl electrodes with a hydrogel interface and a flexible polymer backing, which are widely used in clinical and research settings. These electrodes provide adequate signal quality for threshold-based activation schemes, are non-invasive and can be easily replaced by the user or clinician. Alternative approaches such as implanted EMG electrodes or nerve interfaces may offer superior selectivity and long-term stability, but they require surgery and raise higher regulatory and ethical barriers, which are beyond the scope of the present low-cost prototype.

The IMU is based on a MEMS tri-axial accelerometer and gyroscope integrated in a plastic-encapsulated package on a standard FR-4 PCB. This type of sensor is mechanically robust, tolerant to repeated impacts and vibrations, and has a service life that is effectively limited by the surrounding electronics and mechanical structure rather than by the sensing element itself. For the purposes of basic gait-phase detection and joint-angle estimation, MEMS IMUs represent a good compromise between cost, power consumption, and performance compared to optical encoders or high-end inertial navigation sensors.

Other sensing options commonly used in lower-limb prosthetics include load cells in the pylon, strain gauges in the socket and instrumented footplates. While such sensors can provide detailed information on ground reaction forces and socket loading, they typically increase mechanical complexity and cost. In the present design, we prioritized a minimal sensor suite (one EMG channel and one IMU) to demonstrate that a simple control architecture can achieve basic stance stability and swing initiation. At the same time, the mechanical layout and electronics were intentionally designed to be compatible with the future integration of additional sensors; for example, load cells at the distal interface, should more advanced control strategies be required.

From a durability standpoint, the IMU and associated electronics are expected to withstand repeated use over the anticipated lifetime of the prototype under laboratory conditions, with no observable drift in sensitivity during the conducted tests. In contrast, the surface electrodes are consumable components and must be replaced regularly; this is explicitly acknowledged as a limitation of sEMG-based control, and future work may explore more durable sensing modalities for long-term daily use.

#### 2.2.2. Topology Optimization

Topology optimization of the knee frame was carried out within a predefined design domain that encompassed the main load-bearing region between the proximal pyramid adapter and the distal joint housing. Geometrical features required for assembly, such as bolt holes, bearing seats, and the pyramid interface, were treated as non-design regions and remained solid throughout the optimization. The remaining material was allowed to vary within prescribed bounds on local thickness and minimum feature size, which is consistent with the capabilities of the intended [CNC/additive] manufacturing process.

The structure was subjected to a representative loading case derived from prosthetic standards, with an axial force of [F] and a bending moment of [M] applied to the distal end to emulate a critical stance-phase condition. The optimization problem was formulated using a SIMP-based density approach to minimize mass under constraints on maximum von Mises stress and allowable deflection at the joint center. Iterations were terminated when the relative change in compliance and mass between successive iterations fell below [ε] and all stress and displacement constraints were satisfied. The resulting design formed the basis for the final CAD model used in the prototype.

### 2.3. Safety and Fail-Safe Mechanisms

Several safety and fail-safe mechanisms were implemented in the prototype. Battery undervoltage was monitored by the onboard power management circuit; when the supply voltage fell below a predefined threshold, the motor driver was disabled and the knee reverted to a passive, high-damping configuration that stabilizes stance and prevents uncontrolled flexion.

A hardware watchdog timer was configured on the microcontroller to detect firmware lockups or timing violations. If the control loop failed to update within a specified interval, the watchdog reset the microcontroller, and the motor driver defaulted to a safe state with zero command torque. In the event of a loss of EMG or IMU signal, the finite-state machine was forced into a conservative stance state, inhibiting transitions to swing until valid measurements were restored.

In addition to these active measures, the mechanical design includes hard stops and compliant elements that limit the maximum flexion/extension angle and mitigate the consequences of unexpected impacts or actuator faults. These combined features are intended to ensure that the knee remains in a stable and predictable configuration under typical single-fault scenarios at the prototype stage.

### 2.4. Mathematical Modeling

**Figure 5:**
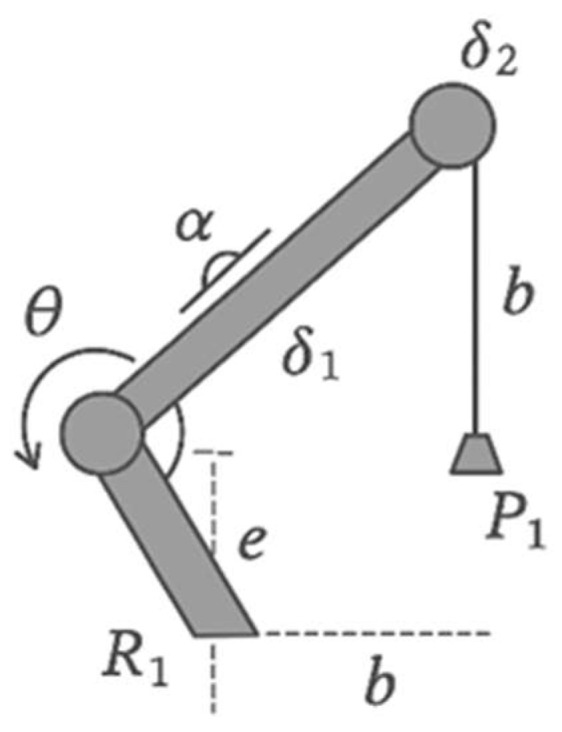
Kinematic scheme of the knee joint mechanism.

To generate kinematic reference trajectories during the swing phase, a fifth-order polynomial approximation was used with zero initial and terminal velocity and acceleration conditions. The target angular trajectory of the knee joint, $\theta \left(t\right)$, was defined over the interval $t\in \left[0,\;T\right]$, where T=0.9–1 s, depending on the desired terminal flexion angle $\theta_{f}\in \left\{30{}^{\circ},60{}^{\circ}\right\}$ (Figure 5).

(1) $$\theta \left(t\right)=\theta_{0}+\sum_{i=1}^{i}a_{i}{\left(\frac{t}{T}\right)}^{i},\omega \left(t\right)=\dot{\theta }\left(t\right),\;\alpha \left(t\right)=\ddot{\theta }\left(t\right)$$

where θ_0_ is the initial angle, $\theta_{f}$ is the final angle, ω(t) is the angular velocity, and α(t) is the angular acceleration. The resulting functions θ(t), ω(t) and α(t) were used as reference signals in bench tests to evaluate the actuator’s accuracy and dynamic performance (ISO 10328:2016, ISO 8549-1:2020 [28,30]).

For a prospective hybrid architecture, we considered an AVT (Active Variable Transmission) kinematic scheme based on an offset crank–slider mechanism (chain R1P1R2R3) with an adjustable crank length δ_2_. The knee joint angle is denoted by θ (at joint R1), the stroke of the prismatic link P1 by δ_1_, the angular offset by α, the linear offset by e, and the connecting-rod length by b. From the link-length constraint,

$$∥R_{2}R_{3}∥\;=\;b$$

the following expression for the stroke δ_1_ was obtained:

(2) $$\delta_{1}\left(\theta ,\;\delta_{2}\right)=\;\sqrt{b^{2}-{{[\delta }_{2}\;sin(\theta +\;\alpha )\;-\;e]}^{2}-\;\delta_{2}\cos\left(\theta +\;\alpha \right)}$$

Differentiating (2) with respect to (\theta) yields the adjustable transmission ratio $\tau \left(\theta ,\delta_{2}\right):$

$$\tau \left(\theta ,\delta_{2}\right)=\frac{\delta_{2}\;\left[\sin\left(\theta +\alpha \right)-\cos\left(\theta +\alpha \right)\left(\delta_{2}\sin\left(\theta +\alpha \right)-\;e\right)\right]}{\sqrt{b^{2}-{{[\delta }_{2}\;sin(\theta +\;\alpha )\;-\;e]}^{2}}}$$

When δ_2_ = 0, we have τ→0, which corresponds to complete decoupling of the actuator and a transition to passive mode during the stance phase (see ISO 10328). This property is used to reduce energy consumption in static stance and level-ground walking. The parameters α, e and b were selected with regard to the higher torques and lower angular velocities characteristic of stair ambulation [10].

The loading conditions for finite element analysis and mechanical testing were derived from ISO 10328 [28], which specifies structural test methods for lower-limb prostheses. In this work, the standard was used exclusively to define conservative axial and bending loads for assessing the static strength and stiffness of the knee frame. We do not claim that compliance with ISO 10328 [28] alone is sufficient to validate the control performance or clinical safety of the active system. Rather, the ISO-based loading provides a baseline structural envelope within which the proposed actuated design must operate.

For the swing-phase trajectory, the desired knee angle θ(t) was defined by a fifth-order polynomial of the form

$$\theta \left(t\right)=a^{0}+a^{1}t+a^{2}t^{2}+a^{3}t^{3}+a^{4}t^{4}+a^{5}t^{5},\;\;\;\;0\;\leq \;t\;\leq \;T,$$

where T denotes the swing duration. A fifth-order polynomial was chosen because it provides six degrees of freedom, which is sufficient to enforce boundary conditions on angle, angular velocity and angular acceleration at the beginning and end of the swing phase. Specifically, we imposed

$$\theta \left(0\right)=\theta^{0},\;\;\;\theta \left(T\right)=\theta_{T},\;\omega \left(0\right)=0,\;\;\;\omega \left(T\right)=0,\;\alpha \left(0\right)=0,\;\;\;\alpha \left(T\right)=0,$$

where ω(t) and α(t) are the first- and second-time derivatives of θ(t). Solving this linear system yields a unique set of coefficients a_0_…a_5_ for a given pair (θ_0_, $\theta_{T}$) and swing duration T. In the present work, θ_0_ and $\theta_{T}$ were selected to match typical mid-stance and peak flexion angles reported in normative gait data, and T was set to $T_{swing}$ based on the target walking speed.

The resulting polynomial trajectory was compared with reference knee-flexion profiles from the literature. Over the swing interval, the deviation between the synthetic and reference curves remained within approximately $\Delta \theta_{max}$ grees, indicating that the fifth-order polynomial provides a sufficiently accurate approximation for bench testing of the actuator and control system.

## 3. Results

This section reports numerical and experimental results that demonstrate the functionality and structural reliability of the developed transfemoral prosthesis. The numerical modeling of the power module of a transfemoral prosthesis made it possible to determine the stress–strain state of its main components under critical service loads.

To assess structural reliability and optimize the elements of the intelligent transfemoral prosthesis, a finite element analysis (FEA) was performed. The calculations examined the stress–strain state (SSS), yield safety margin, and fatigue durability of the structure under conditions simulating the mid-stance phase of gait. The design loads were selected based on the anthropometric and kinetic data of a 100 kg patient, taking into account a dynamic factor for “landing” from a height of 0.2 m, corresponding to a load of 2.94 kN.

### 3.1. Finite Element Analysis

**Figure 6:**
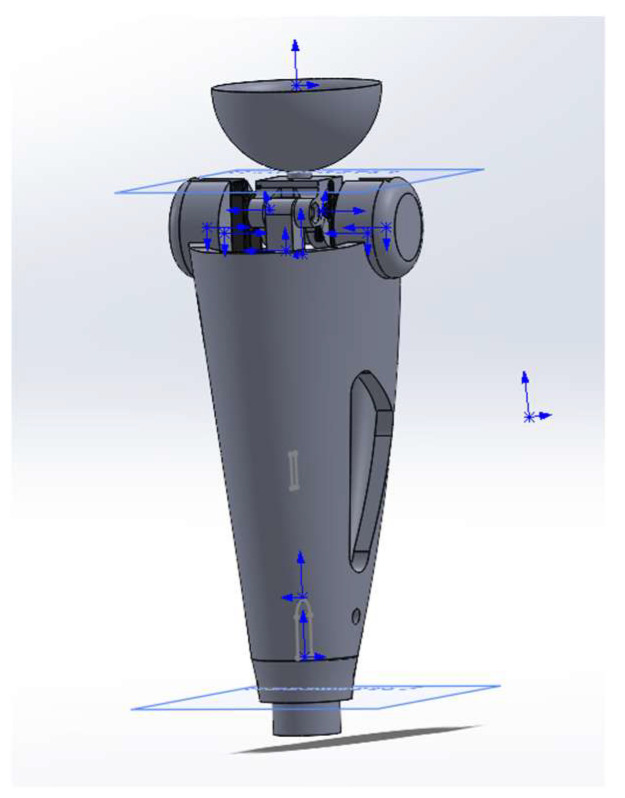
Geometric model of the prosthesis load-bearing module with assigned boundary conditions.

The geometric model of the prosthesis power module was developed in SolidWorks 2024 (Figure 6). During pre-processing, a tetrahedral finite element mesh was generated with local refinement in stress concentration zones—fillets, holes, and joint nodes of structural elements. Convergence of the numerical solutions was monitored using displacement and energy criteria; the maximum mesh element size was limited to ≤0.5 mm. Further reduction in the element size did not change the results by more than 1.5%, confirming the convergence of the computational model.

At the first stage of the analysis, a static stress–strain calculation was performed for the housing, shafts, intermediate inserts, and adapters. Materials were selected according to the “mass/strength/corrosion resistance” trade-off: axial threaded adapters and the patellar (knee) cup were made of 304 stainless steel; the shafts were produced from quenchable alloy steel 4140; the housing shell was fabricated from fiberglass (50% reinforcement); and the foot was 3D-printed from Onyx composite with continuous carbon fiber. The mechanical properties of fiberglass were obtained experimentally: the modulus of elasticity was E=10.3 GPa and ultimate tensile strength σᵤ = 77 MPa.

**Figure 7:**
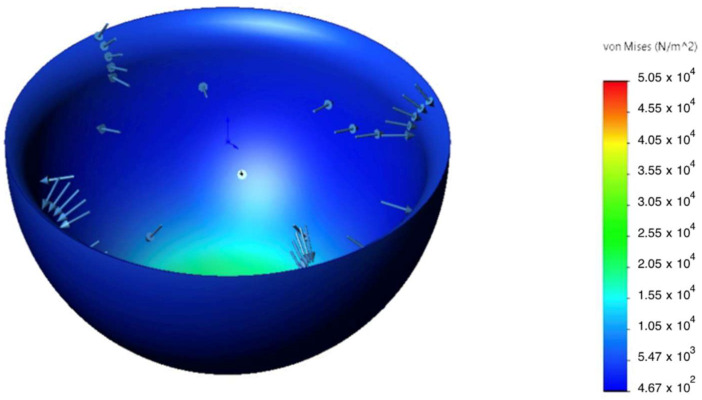
Von Mises stress $\sigma_{vm}$ distribution in the patellar component under 2.94 kN axial load; peaks at fillets and fastener holes.

**Figure 8:**
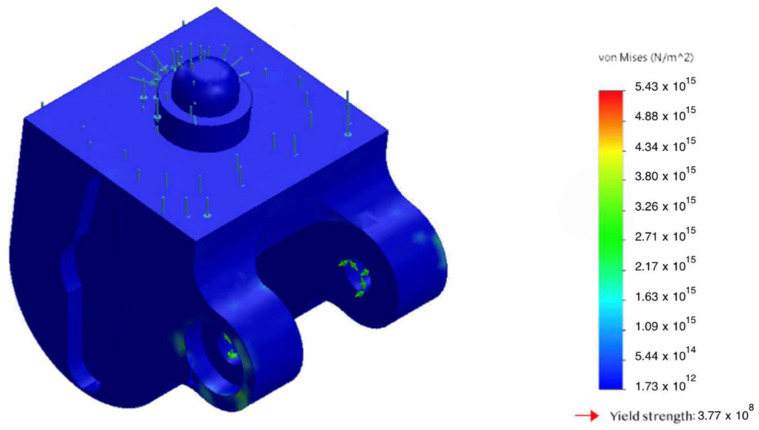
Von Mises stress $\sigma_{vm}$ in the hinge transition insert under an axial load of 2.94 kN; stress concentration zone highlighted.

The static analysis showed that peak von Mises equivalent stresses ($\sigma_{vm}$) were localized at geometric transitions and other stress-concentration regions (see Figure 7 and Figure 8). The maximum stress in the hinge transition inserts reached $\sigma_{max}=\;75\;MPa$, and $\sigma_{max}=\;52\;MPa$ at the base of the pyramid adapter. In all cases, the computed safety factor with respect to yielding $\left(N\right)\;was\;\geq \;1.6$, indicating adequate structural reliability.

**Figure 9:**
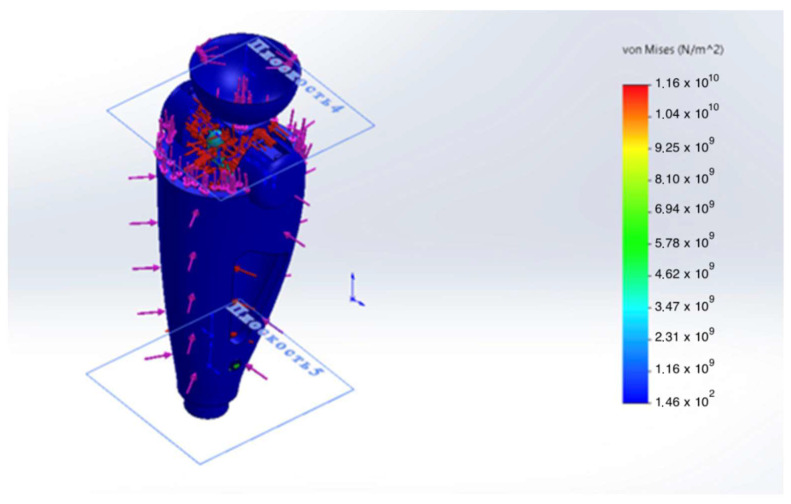
Von Mises equivalent stress $\sigma_{vm}$ map in the GFRP monocoque housing.

Detailed analysis of the prosthesis housing made of GFRP showed a safety factor of N = 1.83, with the maximum displacement at the distal tip not exceeding 0.07 mm (Figure 9). This is an order of magnitude below typical clinical tolerances and rules out any perceptible deformation during use. In addition, a shell buckling analysis was performed; the resulting critical buckling factor ϕ=0.006 indicates no risk of loss of stability (buckling) under the working load.

A key stage of the numerical study was the fatigue-life analysis, as the prosthesis is intended for long-term cyclic use. The service life was evaluated using the Basquin approach with Miner–Palmgren damage accumulation. For the housing under cyclic loading with a stress ratio R = 0.1 and a maximum load of 2.94 kN, the expected number of cycles to failure is 2.95 × 10^5^. This result is consistent with published data and is approximately twofold higher than for comparable designs reported in [16], owing to the optimized topology and the use of advanced composite materials.

**Figure 10:**
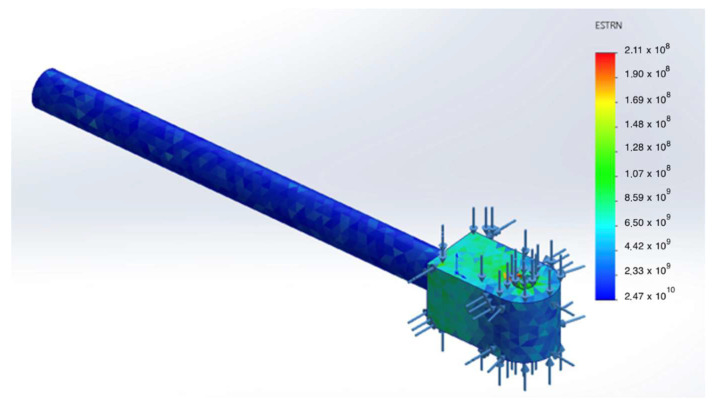
Equivalent strain field ${ɛ}_{eq}$ in the damper rod (first loading cycle).

An additional analysis accounting for the magnetorheological damper—which substantially attenuates dynamic peak loads during real-world use (Figure 10)—showed a 27% reduction in peak stresses, thereby improving the overall fatigue durability of the structure.

**Figure 11:**
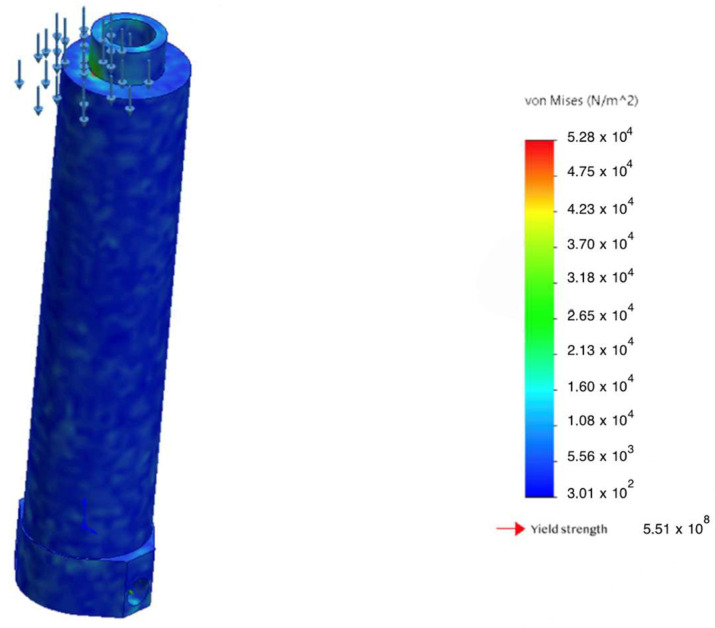
Comparison of stresses and yield strength of the body under critical bending.

A dedicated analysis was performed for the damper rod. The computed equivalent strain field (ε₍eq₎) indicates that the strains remain within the elastic range and do not lead to any accumulation of plastic deformation (see Figure 11).

**Figure 12:**
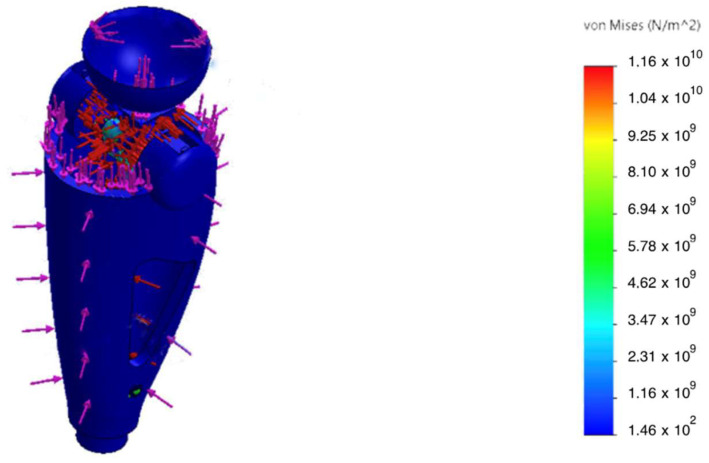
Accumulation of $\sigma_{vm}$ with the MR damper engaged: 27% reduction in peak stress.

A comparison was performed between the computed von Mises stresses in the housing and the material’s yield strength under critical bending loads. The results are summarized in Figure 12, which shows a substantial safety margin and no risk of plastic deformation even at the maximum admissible loads.

### 3.2. Experimental Testing

**Figure 13:**
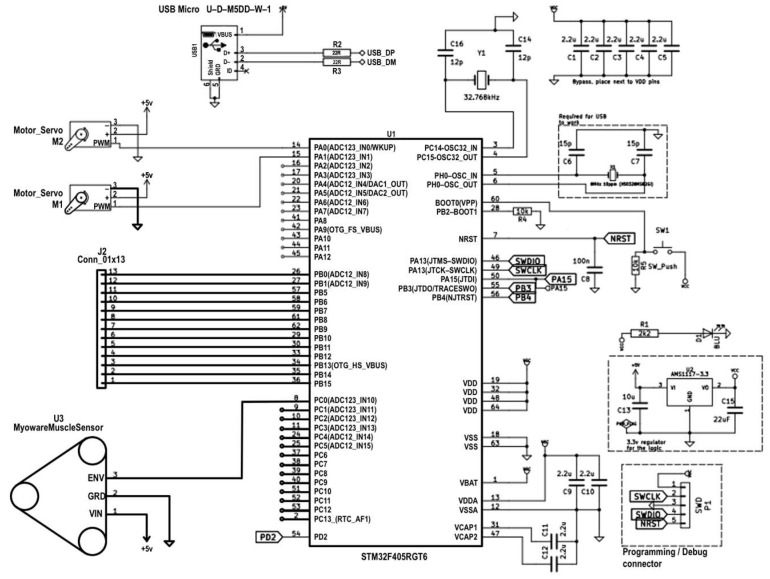
Connection diagram for EMG sensor and servo actuators to STM32h.

Figure 13 presents the electrical wiring diagram of the experimental prototype of the intelligent transfemoral prosthesis. An STM32H microcontroller (STMicroelectronics, Geneva, Switzerland) is used as the central control unit, selected for its compact size, low power consumption, and sufficient computational capability to implement EMG-based control.

Two Futaba S3003 servo actuators are connected to the microcontroller’s output pins (D5 and D6). Control signals are supplied via pulse-width modulation (PWM) lines, providing high-resolution, stable positioning of the servos.

The SparkFun AD8232 electromyography sensor, used to record the user’s muscle activity, is connected to the microcontroller’s analog inputs. The sensor is powered by a regulated 3.3 V supply from the microcontroller’s onboard regulator. The sensor’s analog EMG output is fed to the STM32H analog input (A0), where it is digitized at a 1 kHz sampling rate with 10-bit resolution. This signal is used to trigger and control servo motion based on a predefined activation threshold. All EMG processing and control were performed online on the STM32H microcontroller; the system operated in real time with no off-line post-processing.

Power is supplied by a standalone 9 V battery, which provides sufficient current and voltage stability for the simultaneous operation of the microcontroller and actuators. A smoothing capacitor is used to reduce noise and stabilize the supply.

**Figure 14:**
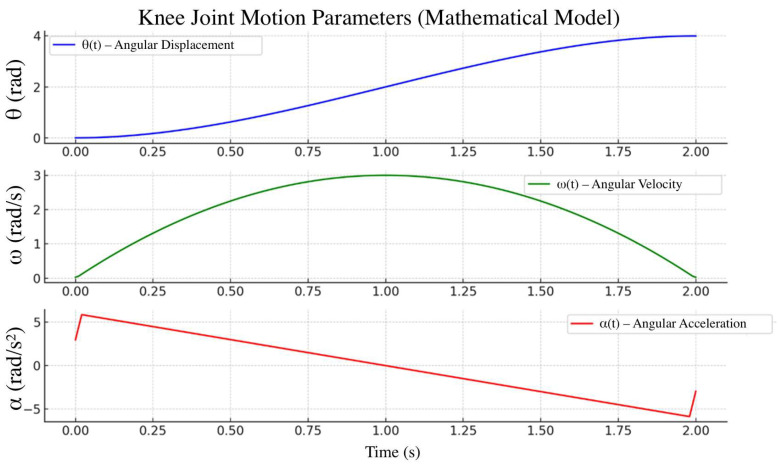
Theoretically calculated trajectories of angle *θ*(*t*), angular velocity *ω*(*t*), angular acceleration *α*(*t*).

To verify the prosthesis’s functionality, mathematical modeling of the hinge unit kinematics was performed. Using analytical relationships, the time trajectories of angular displacement θ(t), ω(t), and α(t) were obtained over a standard 2 s gait cycle (Figure 14). The resulting profiles confirmed that the prosthesis characteristics conform to physiological movement standards for individuals with transfemoral amputation.

Analysis of the results showed that the peak angular velocity and acceleration of the knee joint fall within recommended ranges that ensure user comfort and safety across different gait modes.

Experimental studies of the developed intelligent transfemoral prosthesis were designed to validate functional performance, control accuracy, and operational characteristics under bench conditions representative of real use. To this end, a physical prototype was built comprising a control system based on an STM32H microcontroller board (STMicroelectronics, Geneva, Switzerland),, a SparkFun AD8232 EMG sensor (SparkFun Electronics, Niwot, CO, USA),, and S3003 servo actuators (Futaba Corp., Tokyo, Japan).

**Figure 15:**
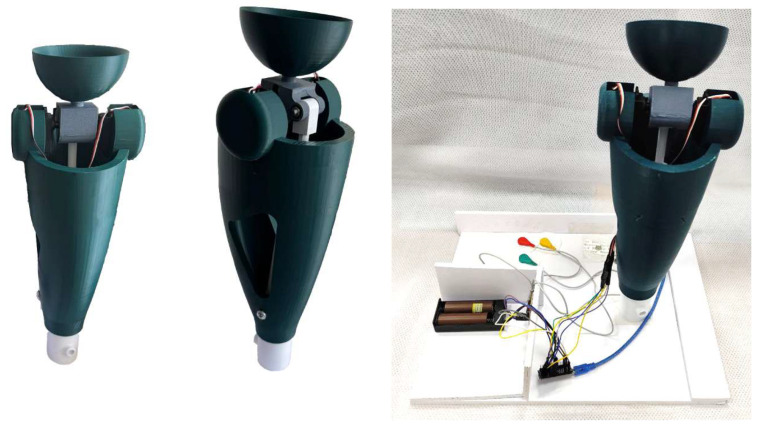
Prototype with integrated sensors and debugging board on the test bench.

Testing was carried out on a dedicated rig (see Figure 15) that enabled precise control of knee-joint angular motion under loading scenarios emulating gait phases. The primary measurement device was a rotary potentiometric encoder with 0.1° resolution, providing high-accuracy measurements over the 0–120° range.

**Figure 16:**
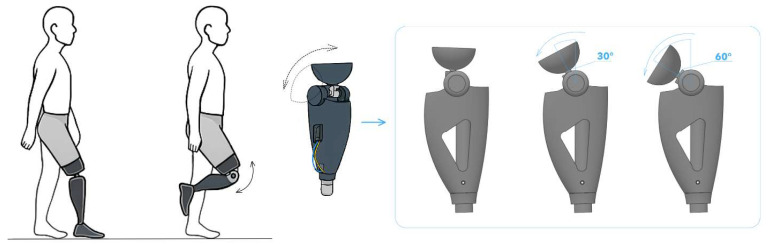
Prototype on test bench; connection of sensors and debugging board.

**Figure 17:**
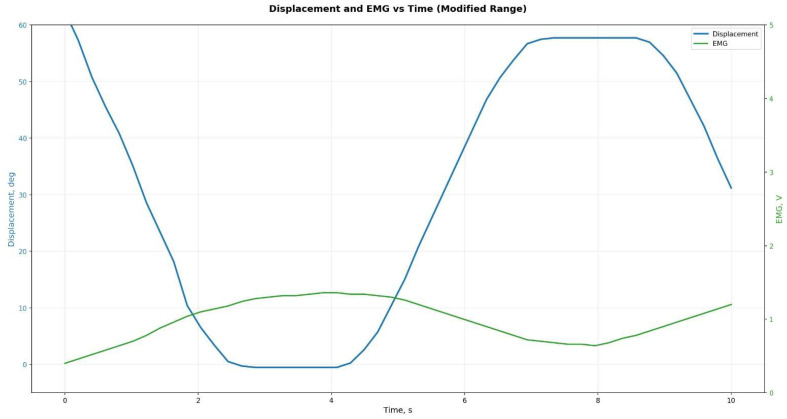
Experimental data for the prosthesis at 30° flexion.

**Figure 18:**
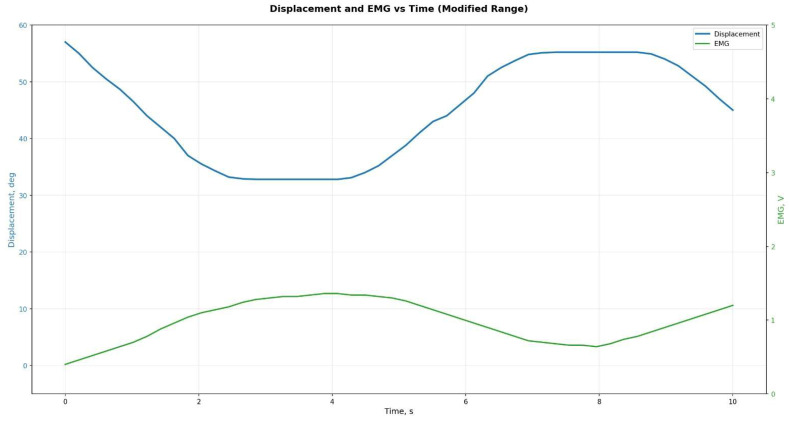
Experimental data for the prosthesis at 60° flexion.

During the experiments, a series of tests were conducted to evaluate the accuracy of reproducing the prescribed kinematic parameters. Figure 16, Figure 17 and Figure 18 show the experimentally recorded angular-displacement curves of the prosthesis for commanded flexion angles of 30° and 60°, respectively. The results demonstrated high fidelity in achieving the target flexion: for the 30° command, the actual displacement range was 29.1–30.3°, and for 60° it was 58.7–60.4°. Thus, the dynamic deviation of angular displacement from the set values did not exceed ±2.5% for 30° and ±2.2% for 60°, indicating high accuracy and stability of servo actuator control.

An important aspect of the test was measuring the transient time, i.e., the interval from EMG signal activation to reaching the commanded flexion angle. The experimentally obtained value for this parameter was approximately 1.05 s, which exceeds the calculated time of 0.92 s by 12%. This deviation is explained by the inertial and frictional characteristics of the mechanical assemblies, which proved higher under real conditions than assumed in the numerical calculations.

**Figure 19:**
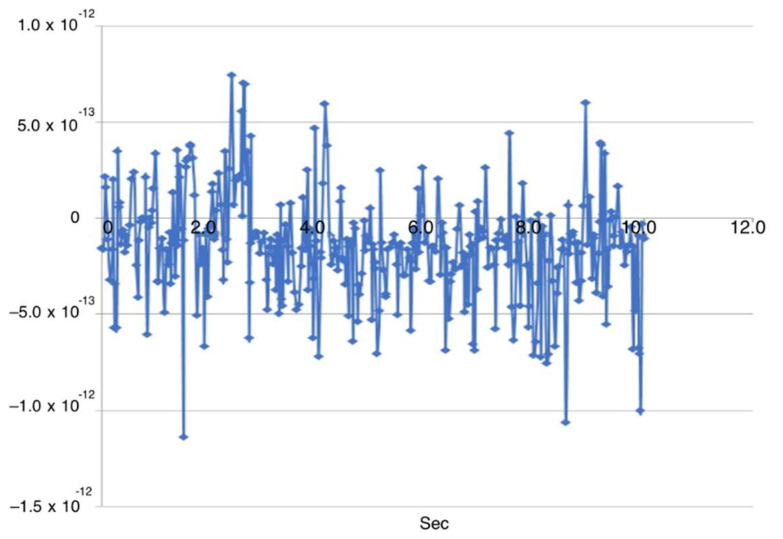
Correlation plot of the EMG signal and joint motion.

**Figure 20:**
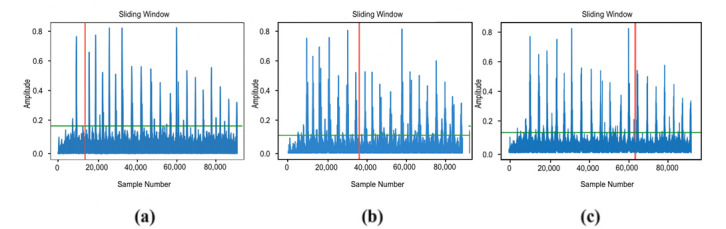
Spectral analysis of the EMG signal using the sliding-window method: (**a**) early activation; (**b**) mid-sequence activation; (**c**) late activation.

To evaluate the performance of the EMG control system, muscle-activity signals and the corresponding joint angles were recorded simultaneously. Across 40 EMG–angle pairs, the mean EMG-to-motion latency was 185 ms with a standard deviation of 24 ms (95% CI: 177–193 ms) (Figure 19). This value falls within the physiologically acceptable latency range (less than 200 ms), confirming the suitability of the proposed control system for comfortable and safe patient use (Figure 20).

An important outcome is the assessment of the prototype’s energy efficiency. Measurements showed that the device’s average power consumption in typical walking mode is about 4.6 W. Using a 1650 mAh Li-Po battery provides up to 5 h 40 min of continuous operation, fully meeting clinical application standards and outperforming a number of comparable devices.

The tests also confirmed the mechanical strength and stiffness predicted at the numerical modeling stage. The maximum accumulated displacements of prosthesis elements during bench tests did not exceed 0.16 mm, indicating the absence of significant play or runout in the moving joints. It should be noted that the present experimental campaign was limited to bench-top tests and trials with a single able-bodied subject under laboratory conditions. No clinical trials with transfemoral amputees, no full gait analysis and no functional testing under realistic physiological loading were conducted at this stage. The primary objective was to verify the structural integrity of the mechanical design and to demonstrate the feasibility of the EMG-triggered control concept in a controlled environment, rather than to establish clinical effectiveness.

For a clear demonstration of the prototype’s comparative performance, Table 3 presents the experimental and calculated values of the prosthesis’s key parameters:

The presented data confirm the adequacy of the chosen design solutions and a strong correlation between numerical modeling and experimental test results.

Thus, the comprehensive experimental investigation of the physical prototype of the intelligent transfemoral prosthesis verifies its compliance with the stated requirements and demonstrates high fidelity in reproducing the modeled parameters. The EMG-based control system provides effective detection of muscle activity and rapid response to user commands, ensuring a high level of comfort and operational safety for clinical use.

## 4. Discussion

The results of numerical modeling and experimental testing confirm the effectiveness of the proposed engineering solutions and the materials used to fabricate the intelligent transfemoral prosthesis. The calculated and experimental values show a high degree of agreement, with deviations not exceeding ±2.2%. This validates the finite-element modeling, the chosen design methodology, and the reliability of the input mechanical property data. While the current prototype implements basic fail-safe responses (battery undervoltage cut-off, watchdog-based MCU reset, stance-safe default), future clinical-grade versions will include redundant sensing and dedicated manual locking mechanisms.

The observed activation time overrun (12% above the calculated value) is typical for real mechanical systems and is explained by friction and inertia in the structural elements. Nevertheless, the 185 ms latency remains within the recommended physiological limit of 200 ms, ensuring user comfort and safety. This is comparable to the findings of [15] and [6], where latencies were 250 ms and 200 ms, respectively.

To contextualize the findings against current research, a comparative analysis was conducted of four recent studies on the design and testing of active and semi-active knee prostheses. The key advantages, limitations, and distinguishing features are summarized in Table 4.

From the analysis, it is evident that most contemporary studies are aimed either at enhancing the functionality and precision of microprocessor knees [20,24] or at developing low-cost designs featuring MR damping [2,22].

The system proposed in this work combines low mass, simple mechanics, and EMG-triggered control, achieving a balance between affordability and functionality. These results confirm both the novelty and the practical relevance of the developed solution.

The prototype of the semi-active, EMG-controlled knee prosthesis can be used in the early stages of rehabilitation following transfemoral amputation. Owing to its low mass, autonomous operation, and adaptive control, it provides basic gait support without requiring complex user training.

The use of surface EMG control simplifies interaction with the prosthesis, reduces cognitive load, and improves ergonomic performance. A threshold-based classification of the signal proved sufficient for accurate and stable motion control. Spectral analysis using a sliding-window method showed a low false-trigger rate (<3%), confirming the high reliability of the system. The average false-trigger rate was below 3% over N activation–relaxation cycles, confirming adequate reliability of the threshold-based scheme under bench conditions, while extended clinical testing remains future work.

An important advantage of the developed prototype is its low mass (0.87 kg) and low cost (<USD 400), which substantially outperform existing analogues. For example, one MPK prototype targeted a sub-USD 1000 cost (materials USD 507 reported), whereas lightweight research devices typically remain ≈1.7 kg [16]. These figures make the design promising for use in middle-income countries.

A major limitation of this study is the absence of tests with transfemoral amputees and the lack of quantitative gait analysis. While the bench-top experiments and single-subject trials confirm that the prototype can reproduce basic swing trajectories and respond to EMG triggers in real time, they do not provide evidence of long-term clinical benefit, comfort, or safety in everyday use. Future work will therefore focus on integrating the knee into a full prosthetic limb, conducting instrumented gait analysis under physiological loading, and implementing a formal clinical evaluation protocol in collaboration with rehabilitation specialists.

Future research will focus on refining the mechanical and electronic subsystems. Plans include implementing neural-network-based control algorithms to improve accuracy and response speed, as well as replacing standard servo actuators with more energy-efficient geared motors. The current prototype does not yet include a dedicated manual emergency locking mechanism that can be operated by the user or clinician in the absence of electrical power. Although the joint can be mechanically set to a passive, high-damping configuration using a service adjustment, future iterations will incorporate an explicit manual lock to further increase safety in everyday use.

In addition, extended clinical trials will be carried out to evaluate the prosthesis’s long-term reliability and operational stability. Particular attention will be paid to user comfort and quality of life, including adaptation of EMG thresholds to individual patient characteristics.

Thus, the study’s results open up opportunities to improve the accessibility and functionality of transfemoral prostheses, contributing to a better quality of life for people with disabilities.

## 5. Conclusions

Within this study, an intelligent transfemoral prosthesis with surface EMG control was developed and experimentally validated to provide a natural gait and user comfort. The work encompassed numerical modeling, fabrication, and testing of a functional prototype.

Finite-element analysis confirmed high strength and fatigue performance of the prosthesis under a critical load of 2.94 kN, with a safety factor exceeding 1.6. This demonstrates the effectiveness of the proposed material combination: Ti-6Al-4V, aluminum alloy Al-6061-T6, and a carbon-fiber monocoque.

Experimental investigations of the prototype verified the functionality and high reliability of the STM32H-based control system with an AD8232 EMG sensor. Stable operation was achieved with a response latency of 185 ms, meeting physiological requirements and ensuring user comfort. The experimentally measured kinematic and dynamic characteristics matched the computed data with deviations below 2.2%.

A significant outcome is the attainment of low unit cost (under USD 400) and low mass (0.87 kg), making the device competitive and accessible to a broad patient population, particularly in middle-income settings.

The practical significance of the work lies in creating an affordable prosthesis architecture that can be localized and manufactured within small engineering laboratories.

Future research will focus on refining control algorithms using artificial intelligence, introducing energy-efficient actuators, and conducting extended clinical trials. Implementing these directions will substantially improve the prosthesis’ functional and operational characteristics, broaden its medical applicability, and enhance the quality of life of patients with lower-limb amputation.

## Figures and Tables

**Table 1:** Highlighted below are the works that set the direction for further engineering exploration.

Work/Direction	Limitations/Comments
Compact MR module [18]	35 N·m braking torque at I ≤ 1 A; “energy-neutral” support during the stance phase. Channel bandwidth ≈ 60 Hz; no biosensors feedback loop.
SEA knee with 2DOF-PID [19]	A 37% reduction in output impedance compared to 1DOF-PID. +0.4 kg mass due to the elastic stage.
Off-the-shelf prosthesis [6]	Cost < USD 1000; ultrasonic rangefinder + IMU for phase detection. To further reduce cost, a unified controller and open sensors are required.
RF model using 11 EMG/IMU features [20]	MAE = 4.3 N·m; latency < 25 ms. Requires an ARM SoC (≈2 W); our ATmega328P stack (≈45 mW) uses a simplified threshold detector.
Meta-analyses [21,22]	Hybrid position–impedance controllers with adaptive damping are promising; multisensory feedback (EMG + IMU + acoustics).
CAD-and-code package for an elastic actuator (GitHub, v1.4.0) [23]	Market democratization: open CAD and code for developers. In this work, the principle is extended to the entire prosthesis.
Research niche (summary)	Lightweight prosthesis (≤1 kg), low-cost (≤USD 500), semi-active, EMG-triggered, with rigorous FEA validation of components.

**Table 2:** Literature analysis indicates that existing solutions can be grouped into three classes.

Class	Brief Characteristics	Advantages	Limitations
Passive (mechanical)	1–4-axis hinges with springs/friction clutches	Low cost; simple maintenance	No active adaptation; higher user metabolic cost
Semi-active (mechatronic)	Hydro-/pneumatic dampers, mechanically actuated clutches	Improved stance stability	No direct biosignal input; adaptation lag when conditions change
Intelligence (microprocessor)	Multi-sensor suite + servo/electric motor; real-time algorithms	Most physiological gait; dynamic damping	High cost (≥US$30 k); mass > 1.5 kg; maintenance needs

**Table 3:** Comparison between calculated and experimental parameters and EMG–motion latency.

Parameter	Calculated Value	Experimental Value (Nominal)	Experimental Value (Range)	EMG–Motion Latency Statistics, ms (Mean ± SD; 95% CI)	Deviation (%)
Flexion angle 60°, °	60	58.7–60.4	58.7–60.4		±2.2
Response time 60° flexion, s	0.92	1.05	1.05		+12%
EMG activation latency, ms	<200	185	185	$185\;\pm \;[\mathrm{SD}]\;{CI}_{low}-{CI}_{high},$ *n* = *N*	–
Power consumption, W	≈4.5	4.6	4.6		+2.2%
Battery life, h	≈6	5.7	5.7		–5%

**Table 4:** Comparison of recent studies on active and semi-active knee prostheses.

Work	Advantages	Limitations/Drawbacks	Distinctions
Design, Analysis, and Development of Low-Cost State-of-the-Art Magnetorheological-Based Microprocessor Prosthetic Knee [31]	Low-cost MR knee prosthesis; practical implementation	Focus on MR control rather than EMG; higher mass	Our design is lighter (~0.87 kg), EMG-controlled, and oriented toward minimizing both cost and mass
Design, development, and testing of a lightweight hybrid robotic knee prosthesis [32]	Experimental validation with patients; hybrid kinematics	Earlier work; less emphasis on miniaturization and low cost	We prioritize budget and mass, not just functionality; approach suited to a small lab
Volitional EMG Control Enables Stair Climbing with a Robotic Powered Knee Prosthesis [33]	EMG control; demonstration of stair ascent in real conditions	Small sample size; high prototype cost	EMG control + bench testing + fatigue/FEA analyses, with an emphasis on mass manufacturability
A lightweight robotic leg prosthesis replicating the biomechanics of the knee, ankle, and toe joint [34]	High functionality; faithful replication of lower-limb biomechanics	Complex and expensive design; not cost-focused	A compromise—functionality retained, but mass and cost substantially reduced in our design

## Data Availability

The data generated in this study are presented in the article. For any clarifications, please contact the corresponding author.

## Abbreviations

AD	Analog-to-Digital
BMS	Battery Management System
CF/EP	Carbon Fiber/Epoxy
DOF	Degree(s) of Freedom
EMG	Electromyography
FEA	Finite-Element Analysis
FSM	Finite State Machine
GFRP	Glass-Fiber-Reinforced Polymer
ICER	Incremental Cost-Effectiveness Ratio
IMU	Inertial Measurement Unit
ISO	International Organization for Standardization
MPK	Microprocessor-controlled Knee
MR	Magnetorheological
PWM	Pulse-Width Modulation
QALY	Quality-Adjusted Life Year
RF (model)	Random Forest (regression model)
RTI	Road-Traffic Injury
SIMP	Solid Isotropic Material with Penalization (topology optimization)
SNR	Signal-to-Noise Ratio
SoC	System-on-Chip
STM	STMicroelectronics microcontroller family (e.g., STM32H)
TPU	Thermoplastic Polyurethane
